# Deciphering the *in vivo* Dynamic Proteomics of Mesenchymal Stem Cells in Critical Limb Ischemia

**DOI:** 10.3389/fcell.2021.682476

**Published:** 2021-06-30

**Authors:** Yipeng Du, Xiaoting Li, Wenying Yan, Zhaohua Zeng, Dunzheng Han, Hong Ouyang, Xiudi Pan, Bihui Luo, Bohua Zhou, Qiang Fu, Dongfeng Lu, Zheng Huang, Zhiliang Li

**Affiliations:** ^1^Department of Cardiology, Zhujiang Hospital, Southern Medical University, Guangzhou, China; ^2^Department of Cardiology, The First Affiliated Hospital of Guangzhou Medical University, Guangzhou, China; ^3^Department of Cardiology, The Second Affiliated Hospital of Soochow University, Suzhou, China; ^4^Department of Bioinformatics, Center for Systems Biology, School of Biology and Basic Medical Sciences, Soochow University, Suzhou, China; ^5^Department of Cardiology, Pinghu Hospital, Health Science Center, Shenzhen University, Shenzhen, China; ^6^Department of Cardiology, Shenzhen Hospital, Southern Medical University, Shenzhen, China

**Keywords:** hind limb ischemia, mesenchymal stem cells, cell therapy, methionyl-tRNA synthetase, proteomics, mass spectrometry

## Abstract

**Objective:**

Regenerative therapy using mesenchymal stem cells (MSC) is a promising therapeutic method for critical limb ischemia (CLI). To understand how the cells are involved in the regenerative process of limb ischemia locally, we proposed a metabolic protein labeling method to label cell proteomes *in situ* and then decipher the proteome dynamics of MSCs in ischemic hind limb.

**Methods and Results:**

In this study, we overexpressed mutant methionyl-tRNA synthetase (MetRS), which could utilize azidonorleucine (ANL) instead of methionine (Met) during protein synthesis in MSCs. Fluorescent non-canonical amino-acid tagging (FUNCAT) was performed to detect the utilization of ANL in mutant MSCs. Mice with hindlimb ischemia (HLI) or Sham surgery were treated with MetRS^mut^ MSCs or PBS, followed by i.p. administration of ANL at days 0, 2 6, and 13 after surgery. FUNCAT was also performed in hindlimb tissue sections to demonstrate the incorporation of ANL in transplanted cells *in situ*. At days 1, 3, 7, and 14 after the surgery, laser doppler imaging were performed to detect the blood reperfusion of ischemic limbs. Ischemic tissues were also collected at these four time points for histological analysis including HE staining and vessel staining, and processed for click reaction based protein enrichment followed by mass spectrometry and bioinformatics analysis. The MetRS^mut^ MSCs showed strong green signal in cell culture and in HLI muscles as well, indicating efficient incorporation of ANL in nascent protein synthesis. By 14 days post-treatment, MSCs significantly increased blood reperfusion and vessel density, while reducing inflammation in HLI model compared to PBS. Proteins enriched by click reaction were distinctive in the HLI group vs. the Sham group. 34, 31, 49, and 26 proteins were significantly up-regulated whereas 28, 32, 62, and 27 proteins were significantly down-regulated in HLI vs. Sham at days 1, 3, 7, and 14, respectively. The differentially expressed proteins were more pronounced in the pathways of apoptosis and energy metabolism.

**Conclusion:**

In conclusion, mutant MetRS allows efficient and specific identification of dynamic cell proteomics *in situ*, which reflect the functions and adaptive changes of MSCs that may be leveraged to understand and improve stem cell therapy in critical limb ischemia.

## Introduction

Peripheral artery disease (PAD) is caused by limited or restricted blood flow in peripheral arteries that can lead to limb pain, disability, or mortality. Critical limb ischemia (CLI) is the most serious form of PAD, which is characterized by a severe blockage in the arteries of the lower extremities and markedly reduced blood-flow (Dua and Lee, 2016; Campia et al., 2019). Cell-based therapies were regarded as an innovative way to improve blood flow recovery in order to prevent necrosis and amputation and limb-threatening complications (Tongers et al., 2014; Cho et al., 2015; Hou et al., 2016). Various stem and progenitor cells have been applied, including whole bone marrow cells, bone marrow mononuclear cells, hematopoietic stem cells, endothelial progenitor cells, and hemangiocytes (Padilla et al., 2007; Zhang et al., 2008; Kwon et al., 2011; Agudelo et al., 2012; Lee et al., 2013). Amid them, mesenchymal stem cells (MSCs) have been regarded as the most potential and promising cell types at both preclinical and clinical levels (Zhang et al., 2018; Jaillard et al., 2020; Yan et al., 2020; Yao et al., 2020).

MSCs are multipotent stem cells with the capacity of self-renewal and multilineage differentiation including mesodermal lineage, endodermal lineage and ectodermal lineage. Besides, they are easy to be obtained and expanded *in vitro*, marking its importance in cell therapy, regenerative medicine and tissue repair (Samsonraj et al., 2017; Trohatou and Roubelakis, 2017; Fu et al., 2019; Zhao et al., 2019). MSCs migrate toward and proliferate in response to chemokine gradients at sites of ischemia, where they aid in the promotion of neovascularization through paracrine effects or by terminally differentiating into vascular cells and myocytes (Lian et al., 2010; Hou et al., 2016; Gu et al., 2017; Shafei et al., 2017; Qadura et al., 2018; Park et al., 2019). However, the specific mechanisms how MSCs respond to and improve the ischemic microenvironment have yet to be fully characterized.

In this study, we utilized a metabolic labeling technique to tag and further enrich nascent proteins in the transplanted cells *in situ* (Erdmann et al., 2015; Sadlowski et al., 2018; Alvarez-Castelao et al., 2019; Han et al., 2020). Mutation of MetRS from L to G at 274 site allowed the utilization of ANL instead Met during protein synthesis. In this way, nascent proteins are tagged with ANL in cells transduced with mutant MetRS (Han et al., 2020). Further identification, enrichment and analysis of ANL tagged proteins will allow us to decipher the dynamic cell functions *in vivo*. We performed mass spectrometry and extensive bioinformatics analysis to ANL tagged proteins in transplanted MSCs in HLI model. The metabolic labeling method doesn’t modify the cells themselves, providing an efficient tool for in-depth and dynamic study of MSCs functions *in vivo*.

## Materials and Methods

### MetRS^L274G^ MSC Transduction and Characterization

Bone marrow-derived mesenchymal stem cells (BMSCs), which were derived from C57BL/6J mice, was purchased from Cyagen Biosciences (Sunnyvale, CA, United States, Catalog Number: MUBMX-01001). (L274G) mutant-mCherry fusion protein was inserted into the cloning site of pMaRSC plasmid (Addgene, Plasmid #89189). BMSCs were seeded at 6 cm dish, and cultured with DMEM media supplemented with 10% FBS. When cells reached to ∼80% confluence, lentivirus was applied with the addition of polybrene (10μg/mL). Seventy-two hours after lentivirus infection, the transduction efficiency in BMSCs was evaluated by mCherry expression. The passage numbers of the MSCs used in all experiments were 6–10.

### Flow Cytometry

Flow cytometry was performed as we previously described (Han et al., 2020). Briefly, BMSCs cells were digested by trypsin and collected by centrifugation. Cells pellet was resuspended in cold PBS and cells concentration was adjusted to 1 × 10^6^ cells/200 μL. mCherry signals were acquired with BDTM LSR II (BD Biosciences, United States) and analyzed by FlowJo software.

### Fluorescent Non-canonical Amino-Acid Tagging (FUNCAT)

MetRS^L274G^-MSCs were cultured in DMEM media containing 10% FBS and 1mM ANL (Jena Bioscience, Jena, Germany) for 24 h. Then cells were gently rinsed with PBS and fixed in 4% formaldehyde in PBS. Click-iT reaction was performed with Click-iT reaction cocktails containing Alexa Fluor 488 alkyne (Click-iT Alexa Fluor 488 Protein Synthesis HCS Assay kit, Life Technologies, Waltham, MA, United States) according to the manufacturer’s instructions. For FUNCAT of MetRS^L274G^-MSCs in the mouse hind limbs, ANL was i.p. administrated at 15 mg/kg every 6 h for 4 times, then the animal was sacrificed. The gastrocnemius muscle was isolated and immediately placed in pre-cooled 4% paraformaldehyde. After overnight fixation at 4°C, the tissue was dehydrated in 30% sucrose for another 12 h and embedded in OCT for preparation of frozen sections. The tissue sections were then evaluated by click-iT reaction as described above.

### Limb Ischemia, Cell Transplantation, and Laser Doppler Imaging

Unilateral high femoral artery ligation and superficial femoral artery (SFA) excision was performed on 6- to 8-week-old male C57BL/6J mice (Jackson Laboratory, Bar Harbor, Me). Hindlimb ischemia surgery was performed as described previously (Niiyama et al., 2009). Briefly, mice were anesthetized with 2% isoflurane in 100% oxygen at a flow rate of 1 L/min. Ligations were placed at the femoral artery and its large branches in C57BL6 mice using 7-0 Prolene (Ethicon, Somerville, NJ) and the femoral artery was excised from its proximal origin as a branch of the external iliac artery to the distal point where it bifurcates into the saphenous and popliteal arteries. Immediately after surgery, the mice were randomly assigned into four groups: the MSC + HLI group, the PBS + HLI group, the MSC + Sham group, the PBS + Sham group. In MSC group, a total of 1.0 × 10^6^ cells in 100 μL PBS was injected intramuscularly into 4 sites of the gastrocnemius muscle. Assessment of limb function and ischemic damage were performed as described previously. Tissue perfusion of the hind limbs was assessed with a laser Doppler perfusion imager (Moor Instruments, Devon, United Kingdom) on days 0, 1, 3, 7, and 14 days after treatment. The digital color-coded images were analyzed to quantify blood flow in the region from the knee joint to the toe.

### HE Staining

The gastrocnemius muscle was isolated and prepared for frozen sections as in FUNCAT. Tissue sections of gastrocnemius muscle from the hind limbs were harvested at different time points. HE staining was performed as described before (Fischer et al., 2008). First, O.C.T. in tissue frozen sections was removed by soaking in ddH_2_O for 5 min. Then the slice was applied into Harris Hematoxyllin solution (Fisher Chemical) for 30 s, then placed in 1% Li_2_CO_3_ (pH 8-8.2, diluted in ddH_2_O) for 30 s after washing with ddH_2_O, then placed in 80% ethanol for 1 min. Eosin was used to stain cytoplasm for 4 min, then dehydrated using 95% EtOH, 100% EtOH twice, and Xylene. At last, the slide was sealed with mounting medium and covered with a coverslip. The images were captured using Olympus IX83 microscope (Olympus, Japan).

### Immunofluorescence

For capillary density analysis, sections were washed with ddH_2_O and PBS. Blocking was performed by incubating sections with 5% BSA in PBST for 1.5 h. Tissue sections were incubated with rabbit polyclonal anti-mouse CD31 (1:100; PA5-38315, Thermo fisher) at 4°C for 12 h. After washing with PBST for three-times, sections were incubated with goat anti-rabbit conjugated with FITC (1:200; Invitrogen) and wash three times to remove free antibody. Then the slices were sealed with mounting medium with DAPI. The images were captured using a laser-scanning confocal microscope, Fluoview FV300 (Olympus, Japan).

### Isolation of ANL-Labeled Proteins From MetRS^L274G^ MSCs-Transplanted Hind Limb

Tissue sections from ischemic and healthy limbs were harvested at days 1, 3, 7, or 14 after HLI surgery, the mice were euthanized and gastrocnemius muscle were collected, ANL was i.p. administrated (15 mg/kg) at every 6 h for 4 times 1 day before collecting muscles. Gastrocnemius muscles were isolated and subjected to Click-iT reaction (Click-&-GoTM Dde Protein Enrichment Kit, click chemistry tools, United States) for enrichment of ANL-labeled proteins on an alkyne resin according to the manufacturer’s instructions. Specifically, cell lysate from different gastrocnemius muscle samples was prepared with RIPA buffer (proteinase inhibitor should be protected from EDTA, which will inhibit click reaction). Then azide-modified proteins were enriched with Click-&-GoTM Dde Protein Enrichment Kit as recommend by manufacturer (Click Chemistry Tools, Scottsdale, AZ, United States). In brief, protein lysate was applied to click reaction with Dde Biotin Alkyne catalyzed with Copper (II) Sulfate. The biotinylated proteins were enriched with streptavidin agarose resin after removing the free Biotin Alkyne. Then peptides from biotinylated proteins were digested by Trypsin and desalt with C-18 desalting cartridges. The digested peptides were concentrated by Speedvac and used for the following MS analysis.

### Sample Preparation and Data Acquisition by LC-MS

Cell Lysate from different samples was prepared with RIPA buffer (proteinase inhibitor should be protected from EDTA, which will inhibit click reaction). Then azide-modified proteins will be enriched with Click-&-GoTM Dde Protein Enrichment Kit as recommend by manufacturer (Click Chemistry Tools, Scottsdale, AZ, United States). In brief, protein lysate was applied to click reaction with Dde Biotin Alkyne catalyzed with Copper (II) Sulfate. The biotinylated proteins were enriched with streptavidin agarose resin after removing the free Biotin Alkyne. Then the peptide from biotinylated proteins will be digested by Trypsin and desalt with C-18 desalting cartridges. The digested peptide will be concentrated using a speedvac. Dried peptides were reconstituted in 20 μL of 0.1% Trifluoroacetic acid. Eight microliter of each sample was injected into a 1260 Infinity nHPLC stack (Agilent Technologies, Santa Clara, CA, United States). This system runs with a Thermo Orbitrap Velos Pro hybrid mass spectrometer, equipped with a nano-electrospray source (Thermo Fisher Scientific, Waltham, MA, United States), and all data were collected in CID mode. The nHPLC was configured with binary mobile phases that included solvent A (0.1%TFA in ddH2O), and solvent B (0.1% TFA in 15% ddH_2_O/85% Acetonitrile), programmed as follows; 10 min at 2% solvent B; 90 min at 5–40% solvent B; 5 min at 70% solvent B; 10 min at 0% solvent B. Following each parent ion scan (300–1200 m/z @ 60k resolution), fragmentation data (MS2) was collected on the topmost intense 15 ions. For data dependent scans, charge state screening and dynamic exclusion were enabled with a repeat count of 2, repeat duration of 30 s, and exclusion duration of 90 s.

### MS Data Conversion and Searches

The XCalibur RAW files were collected in profile mode, centroided and converted to MzXML using ReAdW v. 3.5.1. The mgf files were then created using MzXML2 Search (included in TPP v. 3.5) for all scans. The data was searched using SEQUEST engine, which was set max Missed Cleavages of 5, fragment tolerance of 0.00 Da, parent tolerance of 0.012 Da and variable modifications was + 16 on M (Oxidation), + 57 on C (Carbamidomethyl). Searches were performed by blasting the raw data with the Uniref100 database.

### Peptide Filtering, Grouping, and Quantification

The lists of peptide IDs generated based on SEQUEST (Thermo Fisher Scientific, Waltham, MA, United States) search results were further analyzed using Scaffold viewer (Protein Sciences, Portland, Oregon, United States). Scaffold filters and groups all peptides to generate and retain only high confidence IDs while also generating normalized spectral counts across all samples for the purpose of relative quantification. Protein threshold was set to 99%, minimum Peptides to 2, and Peptide threshold to 80% to be consistent with the cutoff. Scaffold incorporates the two most common methods for statistical validation of large proteome data sets, false discovery rate (FDR), and protein probability. The FDR was set at <1% cutoff, with a total group probability of ≥0.8, with at least two peptides assigned per protein. Relative quantification was performed via spectral counting and spectral count abundances were normalized between samples.

### Bioinformatics and Statistics Analysis

Difference analyses in protein expression between the Sham and HLI groups were performed using a *t*-test, and *p* < 0.05 was taken to indicate statistical significance. 1.5-fold increases or reductions were defined as the standard for differentially expressed proteins (DEPs). Hierarchical clustering was performed to cluster proteins profiles based on complete linkage method. Gene Ontology (GO) analysis were conducted using the interproscan-5 program against the non-redundant protein database (including Pfam, PRINTS, ProDom, SMART, ProSiteProfiles, PANTHER). Functional pathway enrichment of DEPs were performed according to the Kyoto Encyclopedia of Genes and Genomes database (KEGG)^1^. The enrichment pipeline was used to perform the enrichment analysis of GO and KEGG, PANTHER, REACTOME, and WIKI pathway, respectively. A protein-protein interaction network was extracted by matching all the differentially expressed proteins against the STRING-db server^2^, a database of both known and predicted protein-protein interactions.

## Results

### Mutant MetRS Labels Nascent Proteins in the MSCs by Incorporation of ANL

**FIGURE 1:**
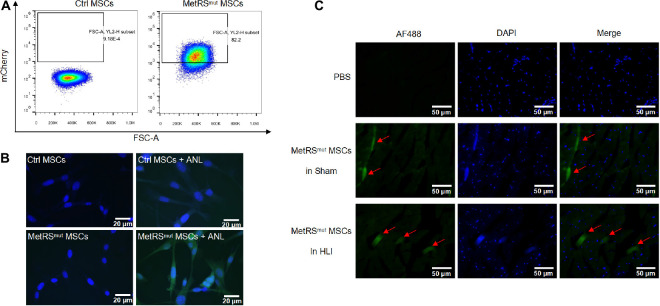
Overexpression of mutant MetRS in MSCs allows tagging of nascent proteins in the cells *in vitro* and *in vivo*. **(A)** FACS analysis of mutant MSCs by the detection of mCherry signal. **(B)** Mutant or control cells were treated with or without ANL followed by FUNCAT analysis. Scale bar, 20 μm. **(C)** Gastrocnemius muscles in HLI and Sham group transplanted with mutant cells or PBS were collected and processed for FUNCAT analysis. Scale bar, 50 μm.

When BM-MSCs at passages 3–4 reached 80% confluence, cells were infected with lentivirus carrying MetRS^L274G^-mCherry as previously described (Han et al., 2020). As shown in Figure 1A, 82.2% of the mutant cells expressed strong mCherry signal and mCherry positive cells were further sorted by flow cytometry. After treatment with 1 mM ANL for 24 h, Fluorescent non-canonical amino-acid tagging (FUNCAT) was performed according to the instructions (Han et al., 2020). Nascent proteins with ANL incorporation in mutant MSCs can be detected by click reaction with alkyne conjugated with Alexa Fluor 488, while no proteins were labeled with Alexa Fluor 488 in control groups, indicating a high labeling efficiency and a good sensitivity of this method (Figure 1B). MetRS^L274G^-MSCs were administered into the gastrocnemius muscle immediately after the surgery, the samples were harvested at days 1, 3, 7, and 14. ANL (15 mg/kg) was i.p. injected every 6 h for 4 times at 24 h before sacrificing the mice. The tissue slices were subjected to click-iT reactions with alkyne-Alexa Fluor 488 (FUNCAT), and the MetRS^L274G^-MSCs showed a strong FUNCAT signal, indicating nascent ANL-tagged proteins in these cells (Figure 1C). Thus, MetRS^L274G^ MSCs can be tagged *in situ* after injection into mouse hind limb by click-iT reaction.

### MSC Treatment Improves Blood Reperfusion in the Mouse Ischemic Hindlimb

**FIGURE 2:**
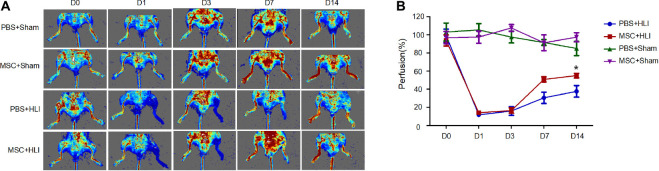
MSC transplantation promoted blood flow recovery in ischemic hindlimbs of mice. **(A)** LDPI measurement of hindlimb blood flow at days 0, 1, 3, 7, and 14 after ischemic surgery, presented as representative images. Colors represent the perfusion degree: red, highest velocity; green, intermediate; blue, low velocity. **(B)** Quantitative analysis of the blood flow perfusion ratio of ischemic-to-non-ischemic hindlimbs at days 0, 1, 3, 7, and 14, respectively (*n* = 3). MSC + HLI, mutant MSCs transplanted to HLI; MSC + Sham, mutant MSCs transplanted to Sham; PBS + HLI, PBS transplanted to HLI; PBS + Sham, PBS transplanted to Sham. *p < 0.05.

**FIGURE 3:**
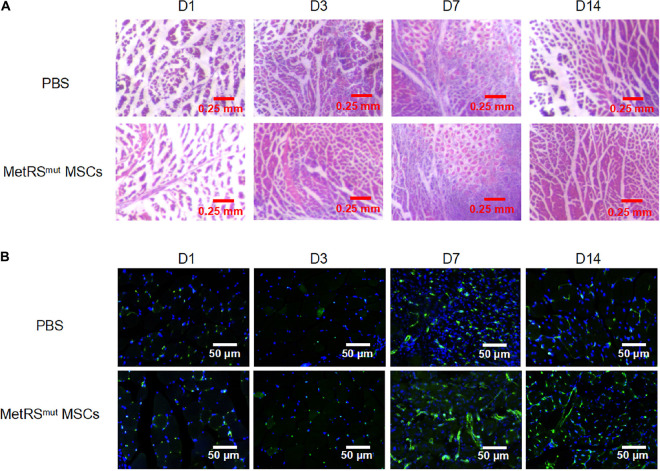
Histological analysis of ischemic hindlimb by HE and vessel staining. Gastrocnemius muscles in HLI group transplanted with either mutant cells or PBS were collected and processed for HE staining **(A**) and CD31 staining **(B)**. Scale bar, 250 μm for **(A)** and 50 μm for **(B)**.

To determine the repair function of MSCs in ischemic tissues, 1 × 10^6^ MSCs in 50 μL of PBS or 50 μL of PBS were transplanted into gastrocnemius muscles immediately after left hind limb ischemia of C57/BL6 mice. Then the blood perfusion was measured by laser doppler perfusion imaging. After ligation, the blood perfusion in left hindlimb severely diminished to ∼10% compared to that of right hindlimb (Figures 2A,B). Mice treated with either MSCs or PBS showed equal proximal and distal blood flow on days 1 and 3 after femoral artery ligation. At day 7, the MSC group showed augmented proximal and distal perfusion, which was much more enhanced at day 14 (Figures 2A,B). Histologic staining were correlated with functional outcomes of blood perfusion in different groups. As shown in the Figure 3A, gastrocnemius muscle fibers in the MSC and PBS groups were seriously disrupted at 1 day after ligation; inflammatory cells were recruited to the ischemic muscle at days 3 and 7 after ischemia. At day 14, the MSC-treated muscles had less inflammatory cells and a relatively intact structure. Angiogenesis was also assessed by CD31 immunofluorescence staining. As shown in Figure 3B, capillary density was severely decreased at day 3 after ischemia. At day 7 after ischemia, vessels became dense where there are the most inflammatory cells. In addition, more larger vessels appeared in MSC group than PBS group at days 7 and 14 after ischemia. In total, the MSC-treated mice showed increased angiogenesis compared with the control mice, which is consistent with the data of blood reperfusion (Figure 2).

### Analysis of the MSC Proteome in the Ischemic Muscle at Different Time Points

**FIGURE 4:**
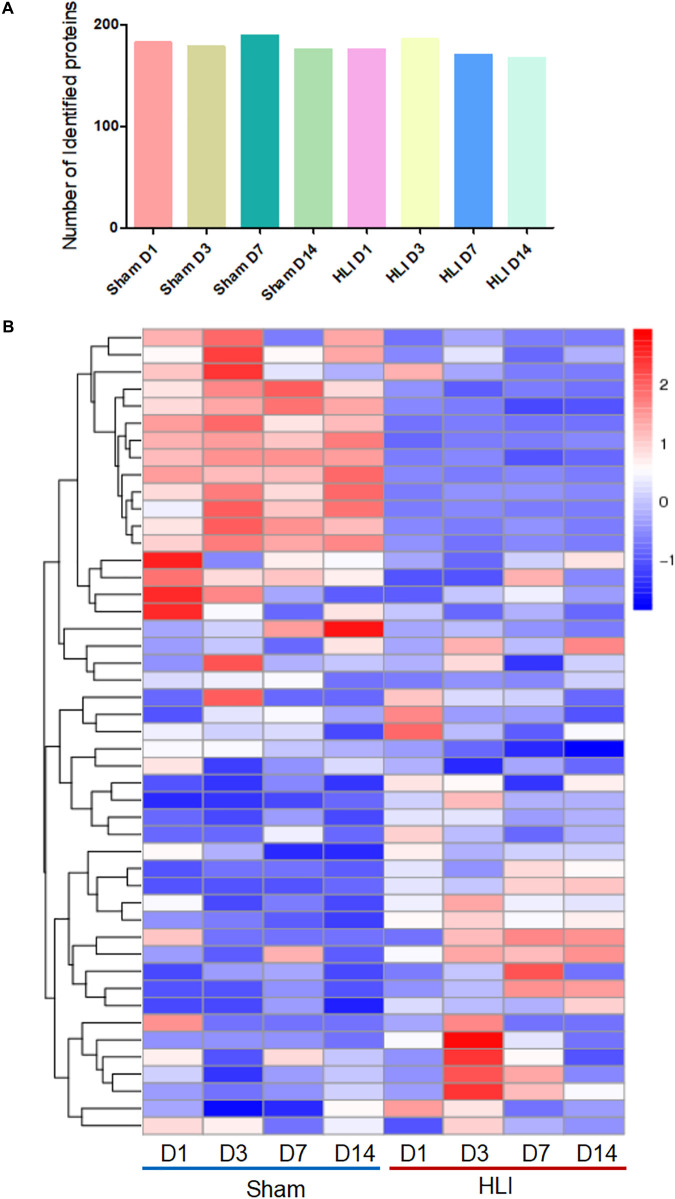
Identification of MSC-specific proteome in the hindlimb. The mice were subjected to HLI or Sham surgery, and mutant cells were i.m. injected in the gastrocnemius muscle. Mice were then randomly grouped and received i.p. injections of ANL at days 0, 2, 6, and 13, gastrocnemius muscles were collect 24 h later for protein enrichment and biological analysis. *n* = 3 mice for each time point in each group. **(A)** Nearly 200 proteins were identified in the eight groups. **(B)** Hierarchical clustering of proteins expressed in all groups. Each column corresponds to the mean expression levels of proteins in 3 independent biological samples, and each row corresponds to a protein.

To delineate the beneficial effects of MSCs in ischemic hindlimb, adaptive changes of MSCs proteomics *in vivo* were detected by highly sensitive click reaction followed by LC/MS analysis. The gastrocnemius muscle lysates, comprised of three biologically independent samples for all groups at each time point, were subjected to isolation of ANL-labeled proteins by a cleavable alkyne-biotin-tag immobilized on streptavidin resin. We obtained ∼4 μg of ANL-labeled proteins from each mouse gastrocnemius muscle transplanted with 1 × 10^6^ MSCs. Notably, a relatively large number of ANL-labeled proteins were identified in the MetRS^L274G^ MSC-injected Sham and HLI muscle at days 1, 3, 7, and 14 post-surgery/cell administration (182, 178, 189 and 175 for Sham and 175, 185, 170 and 167 for HLI, respectively) (Figure 4A). The proteins were analyzed by hierarchical analysis, MSC proteins in HLI group were distinctively different from those in Sham group at four time points (Figure 4B). Moreover, the isolated proteins in HLI group were more pronounced in biological process, such as energy metabolic process and translation (Supplementary Figure 1).

**FIGURE 5:**
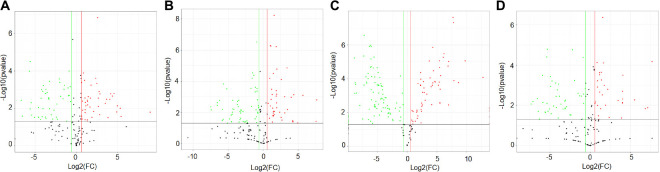
Volcano plot showing the differentially expressed proteins at each time points. Proteins with statistically significant differential expression (≥1.5-fold, *p* < 0.05) at days 1 **(A)**, 3 **(B)**, 7 **(C)**, and 14 **(D)** located in the top right and left quadrants. Red dots indicate up-regulated proteins (ratio > 1.5, *p* < 0.05), green dots indicate down-regulated proteins (ratio < 0.67, *p* < 0.05).

**FIGURE 6:**
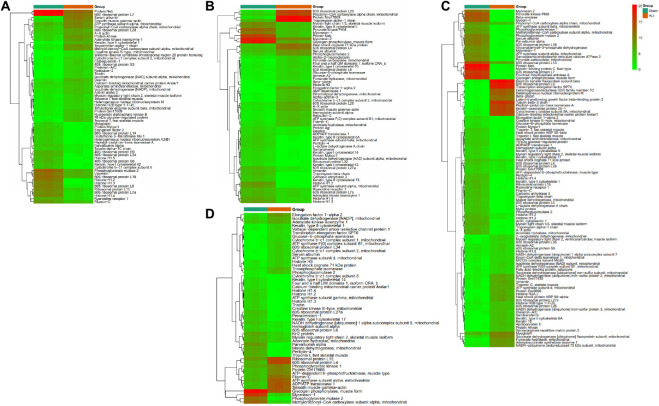
Heat map showing the differentially expressed proteins at each time points. The heat map shows the differentially expressed proteins at days 1 **(A)**, 3 **(B)**, 7 **(C)**, and 14 **(D).** Each row represents a differentially expressed protein and each column, sample replicate. The color scale illustrates the relative level of differentially expressed protein expression: red, higher than the reference channel; green, lower than the reference.

To gather comprehensive information on differentially expressed proteins, we applied the volcano plots for all data in ischemic vs. normal muscle. Interestingly, 34, 31, 49, and 26 proteins were significantly up-regulated, whereas 28, 32, 62, and 27 proteins were significantly down-regulated in HLI vs. Sham group at days 1, 3, 7, and 14, respectively (Figure 5). All differentially expressed proteins at different time points were also displayed by heat map (Figure 6), which give us a better understanding of the functional dynamic MSC proteomes *in vivo*. For example, 60S ribosomal proteins had distinctive expression levels through days 1 to 14, suggesting their different roles at the progressing stages of hind limb ischemia. In addition, cytoskeletal proteins vimentin, A-X actin, and filamin were detected at a higher level in HLI group than in Sham group, suggesting MSCs’ contribution to the production of extracellular matrix in ischemic muscle.

### MSCs Respond to the Ischemic Environment by Altering Proteins Involved in Apoptosis and Energy Metabolism

**FIGURE 7:**
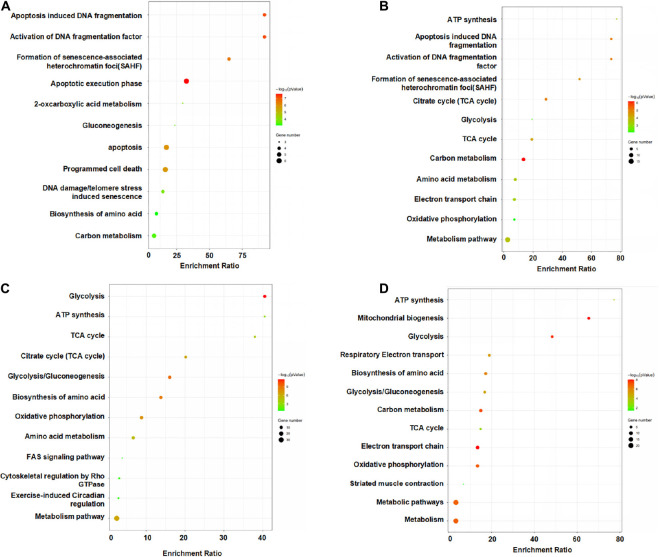
Pathway analysis of the differentially expressed proteins from the MSCs in HLI vs. Sham at each time points. KEGG enrichment analyses of DEPs from the MSCs in HLI vs. in Sham hindlimb at days 1 **(A)**, 3 **(B)**, 7 **(C)**, and 14 **(D)**. KEGG pathway analysis of DEPs indicated a remarkable difference in cellular functions between HLI and Sham. Bubble graphs describe the distribution of DEPs in a classified KEGG pathway. The bubble size represents the differential protein expression level. The red bubbles show that the protein level in HLI is higher than that in Sham, whereas the green bubbles show the opposite trend.

We performed KEGG pathway and enrichment analysis which allows us to identify the enriched biological pathways, and processes involved in the reparative process of MSCs in ischemic hind limb (Figure 7). At days 1 and 3, pathways and proteins related to apoptosis induced DNA fragmentation, activation of DNA fragmentation factor, formation of senescence-associated heterochromatin foci (Sahf), apoptotic execution phase were highly enriched in the HLI group as compared to the Sham group. The highly expressed apoptosis related proteins and pathway indicated a drastic change and adaptation of MSCs in ischemic environment, which can explain the loss of most injected MSCs after injection. At day 7, pathways and proteins related to energy metabolism induced ATP synthesis, glycolysis, TCA cycle, glycolysis/gluconeogenesis, biosynthesis of amino acids, amino acid metabolism were highly enriched in the HLI group as compared to the Sham group. Remarkably, the pathways of energy metabolism gradually possess a dominant position through days 7 and 14. Notably, proteins enriched for apoptosis and energy metabolism are reported to be associated with inflammation and tissue regeneration (Kujoth et al., 2005; Abdel Malik et al., 2017), suggesting that MSC proteomics may be an indispensable resource for ischemic therapy.

**FIGURE 8:**
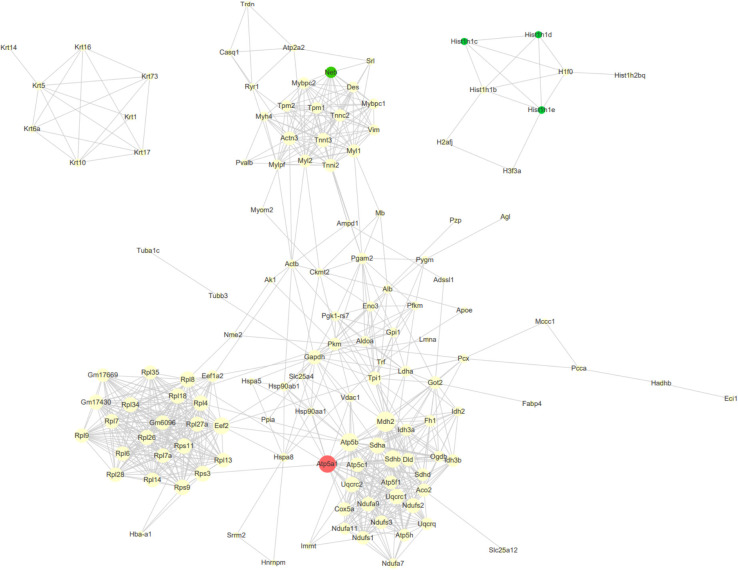
Protein-protein interaction (PPI) network analysis of DEPs. Network analysis using CytoScape of the MSC proteins interactome reveals clustering around nodes involved in apoptosis, metabolism and structural maintenance. The degree of a node indicates the number of connections to other nodes, edge thickness indicates the degree of interactions. The red nodes indicated consistently upregulation DEGs at four time points, and the green nodes indicated consistently downregulation DEGs.

Furthermore, we analyzed protein and protein interaction networks of the differentially expressed proteins from serial time-points (Figure 8) in order to gain an overall understanding of MSC-mediated beneficial effects during ischemic response. MSCs in HLI group expressed an abundance of proteins related to regulation of apoptosis (Atp5a1, Mdh2, Sdhb), which reflects the functional states of MSCs in adaptation to the ischemic environment by maintaining cell survival. In addition, the different proteomic changes of MSCs in HLI were pronounced in ribosome function, protein translation and energy metabolism related proteins (Gm6096, Rpl34, Rpl4, Eef2). Transplanted cells also secreted massive tissue remodeling proteins exerting cytoprotective effects through elevated expression of structure proteins (Mybpc2, Myl2, Myh4, Mylpf) following HLI.

## Discussion

The major findings of this study are: (a) Overexpression of mutant MetRS in MSCs provides a sensitive and specific method to analyze dynamic cell proteomes *in vivo*. (b) The expression of ribosomal proteins were changed at 1 day post-surgery and throughout to 14 days, suggesting disrupted protein translation *in situ* in the ischemic limb; (c) Substantial amount of apoptosis and energy metabolism related proteins were secreted from MSCs and involved in the repair process of HLI.

### Metobolic Labeling of Nascent Proteins *in situ*

MSCs-mediated tissue repair is mainly through releasing growth factors, cytokines, and extracellular vesicles. Potential mechanisms include angiogenesis, beneficial tissue remodeling, anti-inflammatory responses, and finally promotion of tissue endogenous regeneration. However, most MSC proteome studies were performed in cell culture, and the identified factors may not represent their true function *in vivo*. So techniques that could identify cell-type specific proteomes *in situ* may help us better understand how the cells respond to the ischemic microenvironment. Frequently used methods to isolate specific cell types are FACS sorting and laser microdissection of fluorescent labeled cells. Metabolic proteome labeling is a technique that could isolate specific cell proteomes while maintaining cellular integrity during isolation. The first study to perform *in situ* proteome labeling was termed BioOrthogonal Non-Canonical Amino acid Tagging (BONCAT) (Dieterich et al., 2006). In this method, expression of mutant MetRS is driven by a cell-specific promoter. Mutant MetRS could utilize non-canonical amino acid azidonorleucine during protein synthesis. After labeling, click reaction could be performed to biotinylate ANL residues, followed by enrichment via streptavidin beads. In our study, we used the previously established bio-orthogonal proteome labeling method to isolate and analyze cell-specific proteomes in ischemic hind limb.

### MSC Therapy Improved Hindlimb Perfusion in a HLI Model

Intramuscular injection of MSCs increased vascular density and perfusion in the hind limb at 2 weeks post-treatment compared to PBS alone. It is likely that infused MSCs engraft and alter the parenchymal microenvironment and stimulate endogenous regenerative mechanisms. It is consistent with previous studies in mouse HLI model using MSC therapy regardless of stem cell types (allogeneic, autologous, or xenograph), the route of administration (IV, or IM), and the timing of cell transplantation following surgical ligation (Parolini et al., 2010). To investigate cell proteome profiles *in situ* could further reveal targets upon which MSCs alter and decipher mechanisms of action for MSCs in HLI.

### Proteomic Changes of MSCs From HLI in Muscle Tissue

The change of ribosomal proteins to ischemic insults has been shown to persist for up to 14 days. We demonstrated that the increased expression of RPL28 and RPL26 at days 1 and 3 post-surgery, consistent with other reports (Lee et al., 2013; Ong et al., 2013; Han et al., 2020), and elevations declined to normal values by day 14. RPL28 belongs to the ribosomal proteins family. These proteins constitute the large subunit and small subunit of the ribosome primarily responsible for mRNA translation and protein synthesis. In addition to their role in ribosomal biogenesis and protein production, ribosomal proteins possess ribosome-independent functions, especially in tumorigenesis, immune signaling and development. A recent study suggested that high RPL28 was associated with changes in extracellular matrix pathways, indicating a potential role for this protein in tissue remodeling (Labriet et al., 2019; Han et al., 2020). Indeed, we observed an up-expression of several ECM proteins such as filamin-C, and vimentin at different time points after surgery. ECM proteins do not simply serve as structural scaffold that determines the mechanical properties. Under conditions of stress, ECM proteins can drive cell biological responses with an important role in the tissue remodeling (Jourdan-Lesaux et al., 2010; Frangogiannis, 2017; Frangogiannis, 2019). Under hypoxic stress, filamin-C interacted with other molecules to restore cellular membrane integrity, thereby promoting cellular survival during these conditions (Leber et al., 2016; Neethling et al., 2016). Overall, it may be envisioned that ribosomal proteins, apoptosis related proteins and ECM proteins are secreted from MSCs and involved in the repair process of HLI.

There are several limitations of this study. Studies have shown that transplanted MSCs differentiate into endothelial cells, secrete angiogenic factors, and thereby induce neovascularization in ischemic tissue (Al-Khaldi et al., 2003a,b; Kinnaird et al., 2004). In the present study, MSC transplantation markedly increased blood perfusion and capillary density in the ischemic hindlimb compared with control group. However, we didn’t identify many angiogenic factors secreted by MSCs to echo this phenomena. Methionine is the first amino acid of a protein polypeptide, and the signal peptide, which is in the first 16–30 amino acid, is snipped off during translation followed with transport through the endomembrane system. Therefore, secretory proteins lose the first methionine when signal peptide is cleaved off, leading to less ANL incorporation and tagging of secretory proteins. This might explain that secretory proteins are less tagged and enriched in our study. In addition, we did not explore the difference of intramuscular or intra-arterial injections into the ischemic hindlimb that are common routes of cell transplantation. Direct implantation of MSCs has several advantages including increasing the local production of anti-inflammatory and angiogenic factors in the ischemic limb. However, cells remain localized near the site of injection with intramuscular injection, whereas intra-arterially implanted cells distribute broadly in the ischemic tissue, which might differ in their proteomes *in situ*. Further research should determine if different transplantation routes would alter function of MSCs and if some combination of administration routes would be optimal for their therapeutic effects.

## Conclusion

This study provided evidences that overexpression of mutant MetRS in MSCs provides a sensitive and specific method to analyze dynamic cell proteomes *in vivo* in a mouse ischemic hindlimb model. MSCs respond to the ischemic environment by altering proteins involved in apoptosis and energy metabolism. Although we demonstrate a series of protein changes in the ischemic microenvironment, follow-up studies will be required to confirm the proteome profiling in our study.

## Data Availability Statement

The mass spectrometry proteomics data have been deposited to the ProteomeXchange Consortium via the PRIDE partner repository with the dataset identifier PXD026954 and doi: 10.6019/PXD026954.

## Ethics Statement

The animal study was reviewed and approved by the Ethics committee of Southern Medical University.

## Author Contributions

YD, XL, and WY: study design, data acquisition and analysis, manuscript drafting. YD, DH, HO, and XP: data acquisition. ZZ, BL, BZ, QF, and DL: data analysis/interpretation. ZH and ZL: manuscript editing. All authors contributed to the article and approved the submitted version.

## Conflict of Interest

The authors declare that the research was conducted in the absence of any commercial or financial relationships that could be construed as a potential conflict of interest.

## Footnotes

1 http://www.kegg.jp/

2 http://string.embl.de/
