# Supplementary figures and images for: Deciphering the in vivo Dynamic Proteomics of Mesenchymal Stem Cells in Critical Limb Ischemia
Source: Front Cell Dev Biol. 2021 Jun 30;9:682476. doi: 10.3389/fcell.2021.682476 (PMC8278824; doi:10.3389/fcell.2021.682476)

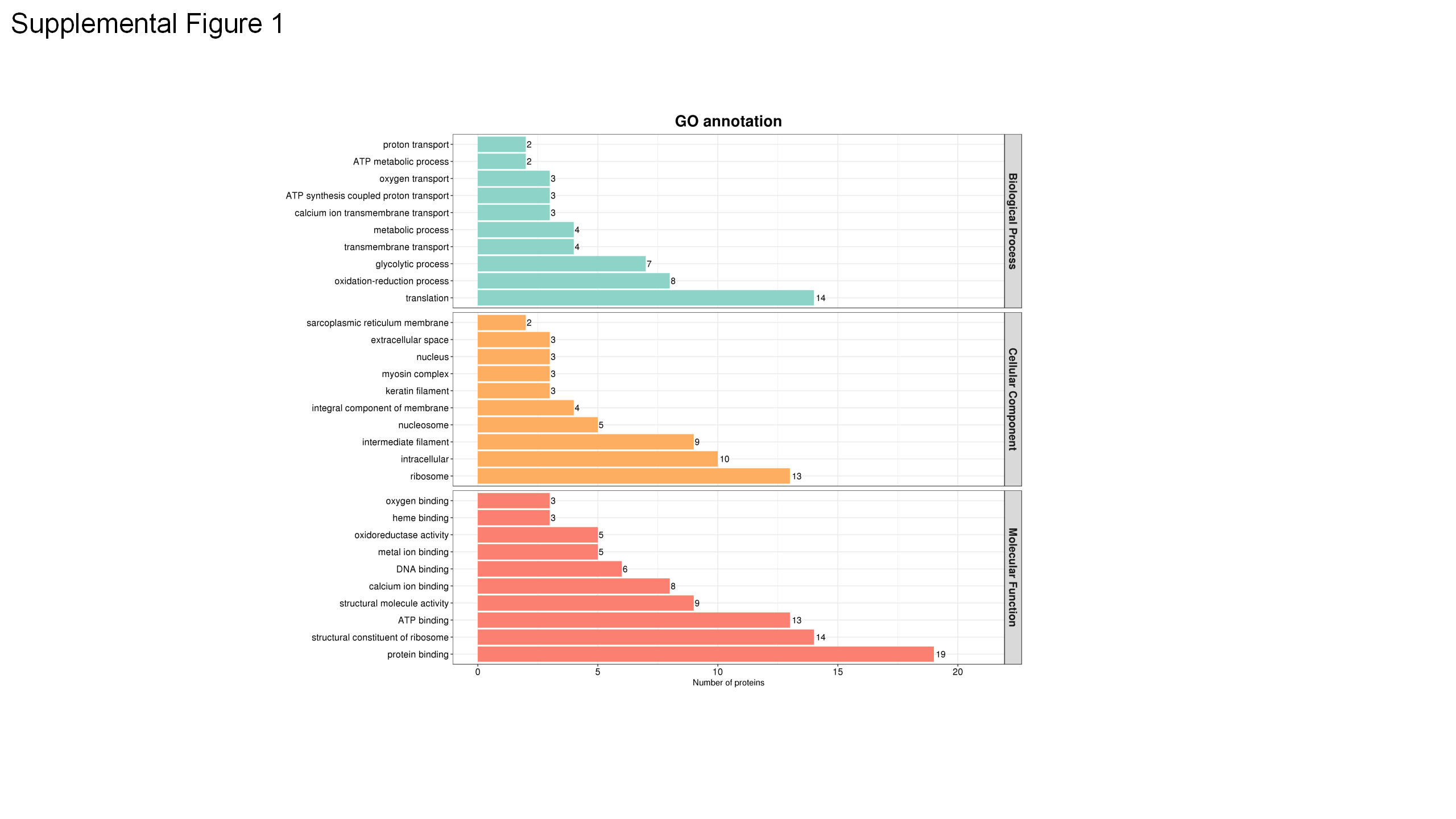

Supplement: Supplementary file 1 [file Image_1.tif]
